# Music's Dual Role in Emotion Regulation: Network Analysis of Music Use, Emotion Regulation Self-Efficacy, Alexithymia, Anxiety, and Depression

**DOI:** 10.1155/2024/1790168

**Published:** 2024-06-28

**Authors:** Min Tan, Xinyu Zhou, Lin Shen, Yonghui Li, Xijing Chen

**Affiliations:** ^1^Key Laboratory of Mental Health, Institute of Psychology, Chinese Academy of Sciences, Beijing 100101, China; ^2^Department of Psychology, University of Chinese Academy of Sciences, Beijing 101408, China; ^3^Faculty of Education, Beijing Normal University, Beijing, China

## Abstract

Music serves as a prevalent emotional regulation tool among young people. However, the correlational and causal relationships between music use, emotion regulation ability, and emotional symptoms remain unclear. This study aimed to investigate the associations and causal relationships between healthy and unhealthy music use, emotion regulation ability, and emotional symptoms, including alexithymia, depression, and anxiety. This study included 16,588 college students nationwide in China. All participants were assessed online with the Healthy-Unhealthy Music Scale (HUMS), the Regulatory Emotional Self-Efficacy Scale (RESE), the Toronto Alexithymia Scale (TAS-20), and the 10-item Kessler Psychological Stress Scale (K10) using a cluster convenience sampling method. We applied a regularized partial correlation network (RPCN) and Bayesian network to analyze the network characteristics of the outcomes. In the RPCN analysis, healthy music use showed the second strongest expected influence (one-step) and correlated positively with emotion regulation self-efficacy while inversely correlating with externally oriented thinking of alexithymia and depression. The Bayesian network indicated that healthy music use was located downstream of the network, positively predicted by managing anger-irritation and expressing positive affect in emotion regulation self-efficacy. In contrast, unhealthy music use in the RPCN displayed the strongest bridge strength and bridge expected influence (one-step). It negatively correlated with expressing positive affect in emotion regulation self-efficacy and positively correlated with alexithymia, anxiety, and depression. The Bayesian network highlighted that unhealthy music use was positively affected by anxiety, depression, and difficulty identifying feelings. In addition, managing despondency-distress influences difficulty identifying feelings through depression, subsequently affecting unhealthy music use and, finally, influencing externally oriented thinking. This study provides a novel framework for understanding the role of emotion regulation self-efficacy and alexithymia in the relationship between music use and emotional symptoms. Emotion regulation and mental health may benefit from music-based interventions and therapies informed by the findings of this study.

## 1. Introduction

Emotion regulation is an important tool for maintaining personal emotional health and happiness in life [1]. It is a process by which people influence what emotions they have, when emotions occur, and how they experience and express emotions [2]. Van Goethem and Sloboda [3] believe that the process of emotion regulation includes four levels: regulation goals, regulation strategies (the process of achieving goals, such as cognitive, behavioral, and avoidance strategies), regulation modes (specific ways to achieve regulation goals), and regulation mechanisms. Based on the four-level process of emotion regulation, the model of emotion dysregulation specifies that an individual's emotional competence influences the setting of emotional regulation goals, thereby determining the regulation strategies and methods adopted by the individual [4].

Emotional competence is the ability to function effectively in emotionally charged environments and includes skills, abilities, and personality traits related to emotion management, such as recognizing and expressing emotions, reacting to others' emotions, and regulating one's own emotions [4, 5]. Emotional competence has a significant impact on the choice of emotion regulation strategies, and the level of emotional competence directly affects the success or failure of individual emotion regulation. Individuals with high emotional competence are more likely to use effective adaptive emotion regulation strategies to achieve beneficial regulation goals, while people with low emotional competence are more likely to adopt maladaptive regulation strategies, leading to emotion dysregulation and preventing the development of emotional competence [4]. Emotion regulation self-efficacy as an important emotional competence refers to an individual's confidence in their ability to effectively regulate emotions and is an important component of emotional competence [5]. It influences the choice of regulation strategies. A study has found emotion regulation self-efficacy positively predicts adaptive coping strategies and negatively predicts maladaptive coping strategies [6], which may exacerbate emotion dysregulation [7]. Based on the model of emotion dysregulation, we propose that emotion regulation self-efficacy may influence people's choice of emotion strategies, such as music use.

Emotion dysregulation refers to emotional experiences and patterns of expression that interfere with objective-oriented activities [8]. It has a significant impact on the development and maintenance of mental health problems and can lead to emotional disorders [9]. Anxiety and depression are two common emotional disorders that are highly comorbid [10] and positively correlated [11]. A study shows that emotion regulation self-efficacy is negatively correlated with anxiety and depression [12]. Alexithymia is an emotional problem characterized by difficulties in recognizing and expressing emotions, with the main symptoms including difficulty identifying feelings, difficulty describing feelings, and externally orientated thinking [13], it is positively correlated with anxiety and depression [14] and negatively correlated with emotional competence. The more severe the symptoms of alexithymia, the worse the individual's ability to express emotions [15]. A study found alexithymia can significantly negatively predict emotion regulation self-efficacy [16]. Self-efficacy in regulating negative emotions can negatively correlates with anxiety and depression [17]. Individuals with anxiety, depression, and alexithymia may have insufficient emotion regulation self-efficacy, which can affect their emotion regulation strategies and effectiveness.

Music is a common method of emotion regulation among young people [18, 19]. Music-induced emotions can affect regions of the brain that affect emotion generation and regulation [20]. Music evokes emotions in a variety of ways, such as activating brain stem reflexes, activating visual imagination, and awakening memories [21]. Research has shown that music can help people effectively regulate their emotions [22, 23]; especially during the COVID-19 pandemic, music is a beneficial tool for emotional regulation and alleviation of loneliness [24]. Research has found that the positive emotions generated when college students listen to music are related to the regulation strategies they use. Those who adopted a cognitive evaluation strategy showed more positive emotions. Relaxation and distraction were the most frequently applied music related strategies by the participants for emotion regulation [25]. Studies also found that positive emotions were more frequent in events with music emotions, such as happiness and joy, compared with negative emotions, such as anger and irritability [26].

Music use as an emotion regulation strategy can be divided into healthy and unhealthy music use [27]. Based on previous research, grounded theory, and three surveys, Saarikallio and colleagues developed the Healthy-Unhealthy Music Scale (HUMS). It categorizes young people's use of music into healthy and unhealthy use by assessing the musical engagement behaviors of them for signs of depressive tendencies from the perspective of depression [28]. Healthy music use refers to an effective and adaptive way of regulating emotions; music is used as a positive means to promote positive emotions and increase interpersonal interaction. Unhealthy music use refers an ineffective and maladaptive way of emotion regulation and itself may be a manifestation of emotion dysregulation [4, 28].The HUMS consists of 13 items divided into healthy (five items) and hnhealthy subscales (eight items), showing good internal consistency and validity [28].

Individuals may show excessive reliance on music to cope with negative emotions or experience negative effects. It includes using music to worsen negative emotions, engaging in rumination or immersion in negative memories in music, or escaping from real life. Research on college students' music use showed that listening to music was positively related to emotion strategies and negatively related to negative emotions. More music listening was related to the more positive role of music in emotion regulation strategy and fewer negative emotions [29].

While adaptive use of music is beneficial, inappropriate or excessive use of music may lead to emotional imbalance and harm individual emotional health [4]. People at risk of illness may combine music with maladaptive behaviors. For example, depressed patients continued to immerse themselves in negative emotions and even felt worse when using music to deal with their emotions [28, 30]. After individuals experienced a mental illness episode, those who changed their music preferences believed that music had damaged their mental state [31]. When young people experienced an episode of mental illness, their interaction with music reflected pathological symptoms [27]. The more severe the symptoms of alexithymia, the stronger the emotional response to music, and the more likely it is to use music to worsen negative emotions [32]. Alexithymia may also affect an individual's ability to perceive emotional aspects of music. Individuals with high levels of alexithymia showed difficulties identifying musical emotions that were inconsistent with their subjective emotions. The difficulty of describing feelings of alexithymia hindered the experience of positive emotions expressed through music [33].

Overall, music may play a dual role in emotion regulation; healthy and unhealthy music use is influenced by emotion regulation self-efficacy. Healthy music use, as an adaptive emotion regulation strategy, can promote positive emotions and improve emotional problems, such as anxiety, depression, and alexithymia. Unhealthy music use, as a maladaptive regulation strategy, can worsen emotional problems. However, there is limited research on the impact of healthy and unhealthy music use on emotion regulation self-efficacy and how they affect emotional problems. Understanding the role of music use in the process of emotion regulation can help people use music for emotion regulation in a more adaptive way and also contribute to relevant intervention methods, such as music therapy, by identifying specific treatment goals and developing a more specialized treatment plan.

Psychological network analysis is a statistical method based on graph theory, which views psychological disorders as networks composed of multiple interacting symptoms [34]. Interactions between symptoms can lead to the occurrence and maintenance of disorders, and there may be shared symptoms between different disorders, leading to comorbidity. Psychological network analysis includes graphical Gaussian model (GGM) network analysis and Bayesian network analysis. GGM networks are made up of variables and their partial correlations, which can reveal the core symptoms of different disorders, the correlations between different symptoms of multiple disorders, and the nodes with greater influence between communities in the network. Bayesian networks are made up of a directed acyclic graph and conditional probabilities of nodes, which can reveal causal influences between symptoms. Typically, the upstream nodes in Bayesian network graphs are considered the main symptoms of the intervention [35].

Based on the model of emotion dysregulation [4], we believe that emotion regulation self-efficacy has an important impact on the choice of healthy or unhealthy music use, and the healthy and unhealthy music use can influence alexithymia, anxiety, and depression. This study takes music use, emotion regulation, self-efficacy, anxiety, and depression as research variables, constructs the GGM network and Bayesian network with healthy music use and unhealthy music use as core factors, explores the promoters of healthy music use, and examines the risk factors of unhealthy music use. The aim of our study was to utilize network analytic methods to explore the associations and potential causation between healthy music use and emotion regulation self-efficacy, alexithymia, anxiety, and depression, as well as between unhealthy music use and these outcomes, respectively.

## 2. Materials and Methods

### 2.1. Participants

This study was approved by the Ethics Committee of Institute of Psychology, Chinese Academy of Sciences (No. H22117), and was conducted from 2022 to 2023 in China. Signed informed consent was obtained from all participants. We conducted an online convenience sampling survey for college students nationwide in China. The research questionnaires were publicly released through an online questionnaire platform. We contacted college teachers from 22 provinces to help distribute the survey to college students. The participants who agreed to participate singed informed consent and then completed the questionnaire.

The inclusion criteria were college students from undergraduate to doctoral level. The exclusion criteria included the college students who could not complete the questionnaire and participants who were identified as non-college students.

### 2.2. Measures

#### 2.2.1. The 10-Item Kessler Psychological Stress Scale (K10)

The levels of anxiety and depression were assessed using the Chinese version of K10 scale [36]. The K10 scale consists of 10 items, with four items assessing anxiety and six items assessing depression. The scale employed a 5-point Likert scale ranging from 1 (almost never) to 5 (all the time). The total score ranges from 10 to 50, with higher scores indicating poorer individual mental health. Based on the scores, individual mental health levels can be divided into four levels: 10–19 (level 1, low risk of mental illness), 20–24 (level 2, relatively low risk of mental illness), 25–29 (level 3, relatively high risk of mental illness), and 30–50 (level 4, high risk of mental illness). The K10 scale includes two subscales that can be scored separately and used to assess individual levels of anxiety and depression. The Chinese version of K10 scale has good internal consistency, with an *α* coefficient of 0.801 [36].

#### 2.2.2. The Toronto Alexithymia Scale (TAS-20)

The TAS-20 was used to assess the severity of participants' alexithymia. The TAS-20 scale consists of 20 items. Each item is scored on a 5-point Likert scale, ranging from 1 (completely disagree) to 5 (completely agree). TAS-20 includes three dimensions: difficulty describing feelings (DDF), difficulty identifying feelings (DIF), and externally oriented thinking (EOT). TAS-20 can be scored as a total score or separately for each dimension. The total score of TAS-20 ranges from 20 to 100, with a cutoff score of 0–51 (no alexithymia), 52–60 (possible alexithymia), and 61–100 (alexithymia present). The highest average subtype score indicates what part of alexithymia the individual has the greatest challenge with. The Cronbach's alpha coefficient of TAS-20 is 0.945 [37].

#### 2.2.3. The Regulatory Emotional Self-Efficacy (RESE) Scale

The RESE Scale was used to assess participants' emotion regulation self-efficacy. The scale consists of three dimensions: expressing positive affect (POS), managing despondency-distress (DES), and managing anger-irritation (ANG). POS means self-efficacy in expressing positive affect, while DES and ANG mean self-efficacy in managing two different negative affects which representing self-efficacy in managing negative affect. Each dimension includes four items, with each item scored on a 5-point Likert scale, ranging from 1 (very poor) to 5 (very strong). The scale is scored separately for each dimension, with scores ranging from 5 to 20. Higher scores indicate a better corresponding emotion regulation self-efficacy. The scale has good internal consistency, with a Cronbach alpha coefficient of 0.82 [38].

#### 2.2.4. The Healthy-Unhealthy Music Scale (HUMS)

We used the HUMS to assess participants' music use. HUMS categorizes music use into healthy music use (HMU) and unhealthy music use (UHMU) based on users' genuine feelings and specific emotional experiences while using music. HMU includes improving mood, reassessing, and diverting attention, while UHMU includes rumination through repeated listening, using music to escape and avoid problems and distressing relationships, and experiencing a worsening trend toward sadness, anger, negative energy, and depression through listening to music [28]. The HUMS scale consists of 13 items, including 5 items for HMU and 8 items for UHMU. Each item has five options, rated on a 5-point Likert scale ranging from 1 (never) to 5 (always). The scale is scored separately for each dimension, with higher scores indicating more frequent use. In this study, the Cronbach's alpha coefficient for this scale is 0.827.

### 2.3. Data Analysis

#### 2.3.1. Descriptive Statistics

We used statistical IBM statistics 27.0 to perform the statistical analyses of the participants' demographic outcomes, such as gender, age, education level, and the music learning experiences, as well as the mean and standard deviation of the measures.

#### 2.3.2. Network Analysis

We calculated the standard scores of each dimension of the K10, the HMUS, the RESE, and the TAS-20 and then used them in the network analysis.

#### 2.3.3. Regularized Partial Correlation Network (RPCN) Analysis

For our network analysis, we used the R programming language, version 4.3.1 [39]. We first constructed a partial correlation network using graphical Gaussian model (GGM) [40]. The GGM is used to evaluate the network model when the observed variables are continuous data. Within the GGM Network, nodes symbolize the variables under evaluation, while undirected edges denote the inter relationships among these nodes. The color of the edges signifies the correlation polarity between nodes: typically, red indicates a negative correlation, while green or blue suggests a positive correlation. The thickness is indicative of the strength of the association between nodes. Data normalization to a Gaussian distribution was achieved using the nonparanormal transformation method, as implemented in the huge package [41, 42]. To refine the GGM model, we applied the Lasso algorithm, which serves to exclude the inclusion of tenuously correlated edges within the network graph [43]. The construction of the network graph was facilitated by the qgraph package, version 1.9.5 [44].

We used the estimate group network method from the bootnet package (version 1.5.4) to calculate node centrality indices [45]. These indices are critical for evaluating the prominence of each node within the network and include the centrality of strength, closeness, and betweenness centrality. Strength centrality is the aggregate of the weights of all direct connections to a node, indicating the node's level of connectivity within the network. Closeness centrality is determined by the sum of the inverse weights of the shortest paths from a node to all other nodes, signifying the node's accessibility and its capacity for rapid influence dissemination throughout the network. Betweenness centrality measures the extent to which a node acts as a bridge along the shortest path between two other nodes, highlighting its role in controlling the flow of information within the network [40].

We used the bridge method from the network tools package to compute the bridge centrality indices of the nodes [46]. Bridge centrality assesses the influence between different communities within a psychological network. Bridge centrality indices are crucial for pinpointing key nodes that serve as connectors between distinct communities, thereby indicating the nodes' capacity to mediate interactions between these groups. Psychological networks typically consist of communities that aggregate nodes with similar characteristics. These nodes often correspond to various symptoms of a particular disorder or to multiple facets of a single psychological scale. Bridge centrality indices encompass four distinct measures: bridge strength, bridge closeness, bridge betweenness, and bridge expected influence (one-step). Bridge strength is defined as the aggregate weight of all edges that link a node to nodes in other communities. Bridge closeness is calculated as the inverse of the mean path length from the node to the nodes in other communities. Bridge betweenness quantifies the frequency with which a node occurs on the shortest paths connecting nodes across different communities. Lastly, bridge expected influence (one-step) is the sum of the weights of edges directly connecting a node to nodes in other communities, which serves as an indicator of the node's immediate impact on these nodes.

We evaluated the resilience of centrality measures within network nodes. Initially, confidence intervals for each edge weight were computed to determine the precision of the network graph regarding the disparities in edge weights. Subsequently, the robustness of centrality measures was gauged by resampling of data subsets via the bootstrap technique. Rapid alteration in centrality measures with a decreasing sampling ratio indicates reduced stability [40, 41].

#### 2.3.4. Bayesian Network Analysis

Bayesian networks are structured as directed acyclic graphs (DAGs), consisting of nodes and their conditional probability distributions [47]. These networks facilitate the identification of causal relationships among nodes and the quantification of their respective probability distributions. Unlike the RPCN, Bayesian networks feature directed edges, which signify causal influences; for instance, an edge from A to B (A→B) denotes that A is the cause and B is the effect, implying that A induces B and A is B's parent node.

To construct the Bayesian network (BN), we employed the hill-climbing (HC) algorithm available in the bnlearn package (version 4.9) within the R programming environment [48]. We firstly estimated the optimal Bayesian network by restarting the HC algorithm 50 times and applying 100 perturbations during its search process. Then we used bootstrapping method to find the most stable BN among different numbers of samples; bootstrap resampling can reliably assess the edges in the BN are reliably supported by the data. By drawing 1,000 random samples from the data, we generated the corresponding Bayesian networks for each sample. Postsampling, we computed the frequency of occurrence for each edge; a higher frequency indicates a stronger likelihood of the edge's presence in the network. Following this, we constructed an averaged network consisting with edges appearing in over 85% strengths and 50% directions of the fitted networks. Finally, we used the strength.plot function to output the network in descending order of edge strength [49].

After constructing the averaged Bayesian network, we used lm method to fit linear model of each child node within the averaged Bayesian network [50]. Lm uses the method of least squares, which can find the best fitted regression line that best fits the given dataset [51], which visualized in the averaged BN. The results of lm method include the total effects of the linear model, and direct effects of each parent node on the child node. The parameters of the total effects include *R* square, residual standard error (RSE), and *P* value, and the parameters of direct effects include the intercept, estimate (*β*), and standard error (SE) and *P* value. The intercept represents the value of a given node when all its parent nodes are set to zero. The *β* suggests the change in the child node's value per unit change in the parent node, with positive values denoting reinforcement and negative values indicating attenuation. The SE represents the standard deviation of the sampling distribution of a statistic, indicating the average variability around true value of the parameter being estimated.

## 3. Results

### 3.1. Descriptive Statistics

A total of 16,772 participants filled out the survey, and 16,588 valid data were collected after deleting duplicate data and those surveys not being filled out by college students, resulting in a valid questionnaire response rate of 99%. The mean age of 16,588 subjects was 19.82 ± 1.78 years old. There were 9,128 male students (55%) and 7,460 female students (45%), among which 13,378 were vocational college students (80.6%), 2,550 were undergraduate students (15.4%), 520 were graduate students (3.1%), and 140 were doctoral students (0.8%).Four thousand nine hundred seventy-seven (30%) of them had music learning history, and 11,611 (70%) subjects had no history of music learning.  Table 1 shows the mean scores and standard deviations of each measurement for the whole sample and for each gender.

### 3.2. Network Analysis

To make the diagrams more concise, we used the abbreviations of the variables in the RPCN and Bayesian network analyses (Table 2).

### 3.3. Networks of Healthy Music Use

#### 3.3.1. The RPCN of Healthy Music Use

**Figure 1:**
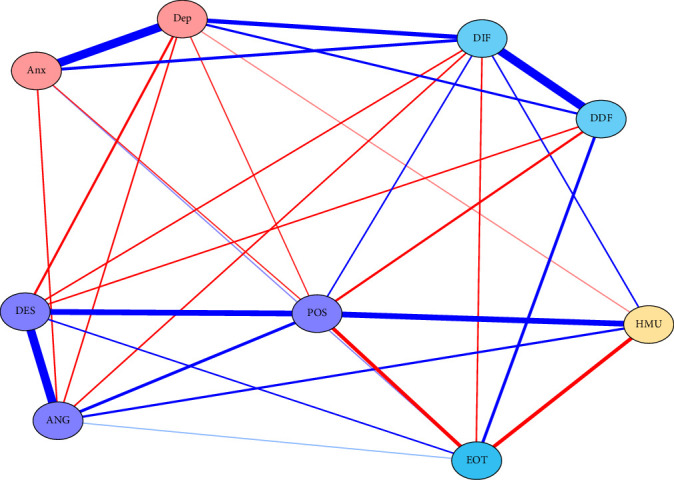
The regularized partial correlation network of healthy music use.

In the RPCN of healthy music use (Figure 1), healthy music use was positively correlated with expressing positive affect in emotion regulation self-efficacy and negatively correlated with externally oriented thinking in alexithymia. The three dimensions of emotion regulation self-efficacy were positively correlated. In alexithymia, externally oriented thinking was negatively correlated with difficulty identifying feelings and positively correlated with difficulty describing feelings; difficulty describing feelings was positively correlated with difficulty identifying feelings. Expressing positive affect in emotion regulation self-efficacy was negatively correlated with externally oriented thinking in alexithymia. Managing despondency-distress and managing anger-irritation in emotion regulation self-efficacy were negatively correlated with depression.

**Figure 2:**
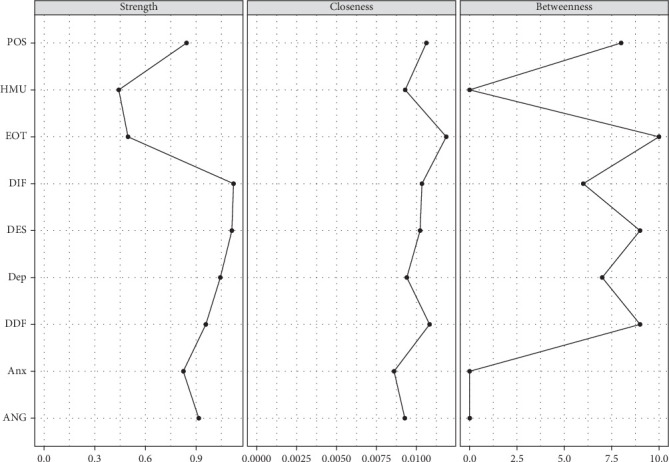
Node centrality indices of the regularized partial correlation network of healthy music use.

Figure 2 shows the node centrality indices of RPCN of healthy music use; the top three strength nodes in strength were difficulty identifying feelings, managing despondency-distress, and depression. The top three nodes in closeness were externally oriented thinking, difficulty describing feelings, and expressing positive affect. The top three nodes in betweenness were externally oriented thinking, managing despondency-distress, and difficulty describing feelings.

**Figure 3:**
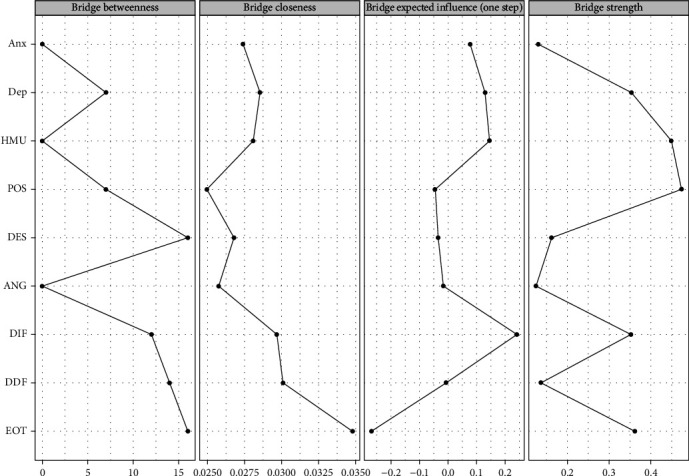
Bridge centrality indices of the regularized partial correlation network of healthy music use.

Figure 3 shows the bridge centrality of the RPCN of healthy music use; the top three nodes for bridge strength were expressing positive affect, healthy music use, and externally oriented thinking. The top three nodes for bridge closeness were externally oriented thinking, difficulty describing feelings, and difficulty identifying feelings. The top three nodes for bridge betweenness were externally oriented thinking, managing despondency-distress, and difficulty describing feelings. The top three nodes for bridge expected influence (one-step) were difficulty identifying feelings, healthy music use, and depression.

The CS coefficients of the bootstrapping test result for node centrality indices were strength (CS (cor = 0.7) = 0.75), closeness (CS (cor = 0.7) = 0.75), betweenness (CS (cor = 0.7) = 0.75), all reaching the highest CS-C level of 0.75, indicating that the network was very stable [41, 42]. The figure of centrality stability and that of bootstrapped confidence intervals of estimated edge weights are attached in the supplementary materials (*Supplementary 1*: Stability of the centrality metrics of the RPCN of healthy music use. *Supplementary 2*: Bootstrapped confidence intervals of estimated edge-weights for the RPCN of healthy music use).

#### 3.3.2. The Bayesian Network of Healthy Music Use

**Figure 4:**
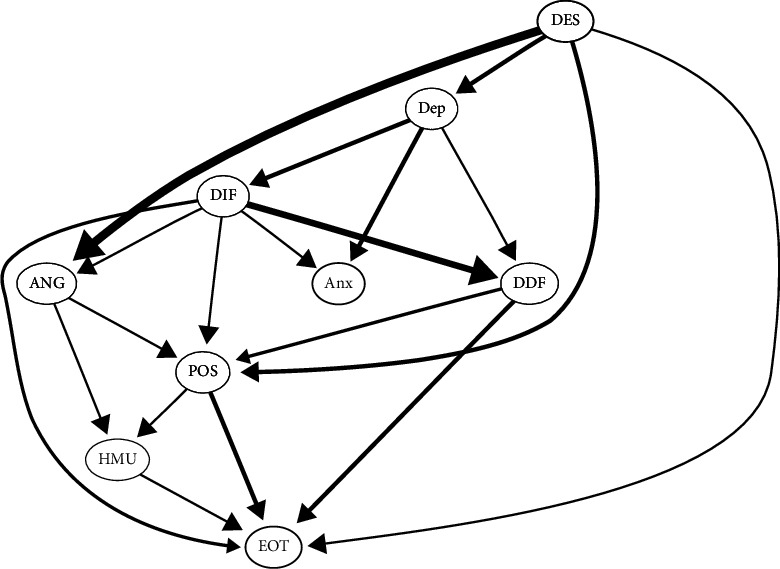
The averaged bootstrapped Bayesian network of healthy music use. This figure shows the averaged Bayesian network generated by bootstrapping sampling data of healthy music use Bayesian network data. The probability of the edges appearing in the graph is not less than 85%, and the probability of the edges pointing is not less than 50%. The thickness of the edges represents the strength of the influence between nodes; that is, the thicker the edge, the greater the degree of influence between nodes. These edges are sorted from high to low according to their influence strength.

The averaged bootstrapped Bayesian network of healthy music use (Figure 4) has the following characteristics: managing despondency-distress was at the top of the network. The nodes that can be directly influenced by managing despondency-distress were managing anger-irritation, depression, expressing positive affect, and externally oriented thinking. Healthy music use was downstream in the network; it was influenced by managing anger-irritation and expressing positive affect and can influence externally oriented thinking. Managing despondency-distress affected difficulty identifying feelings or difficulty describing feelings of alexithymia through depression, then affected managing anger-irritation or expressing positive affect, then affected healthy music use, and finally influenced externally oriented thinking.

**Figure 5:**
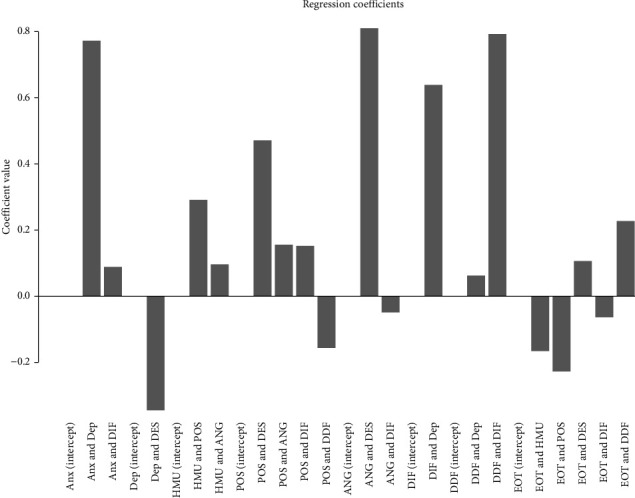
Regression coefficients of each child node of bootstrapped averaged Bayesian network of healthy music use.

Figure 5 shows the regression coefficients of each child node within the averaged bootstrapped Bayesian network of healthy music use. This figure has the following characteristics: depression was negativily predicted by managing despondency-distress. Anxiety was positively predicted by depression and difficultly identifying feelings. Healthy music use was positively predicted by managing anger-irritation and expressing positive affect. Externally oriented thinking was negative predicted by healthy music use, expressing positive affect, difficultly identifying feelings, and positively predicted by managing despondency-distress and difficulty describing feelings. You can see more details of the linear model in the supplementary materials (*Supplementary 3*: The regression coefficients of each child node in the averaged bootstrapped Bayesian network of healthy music use).

### 3.4. Networks of Unhealthy Music Use

#### 3.4.1. The RPCN of Unhealthy Music Use

**Figure 6:**
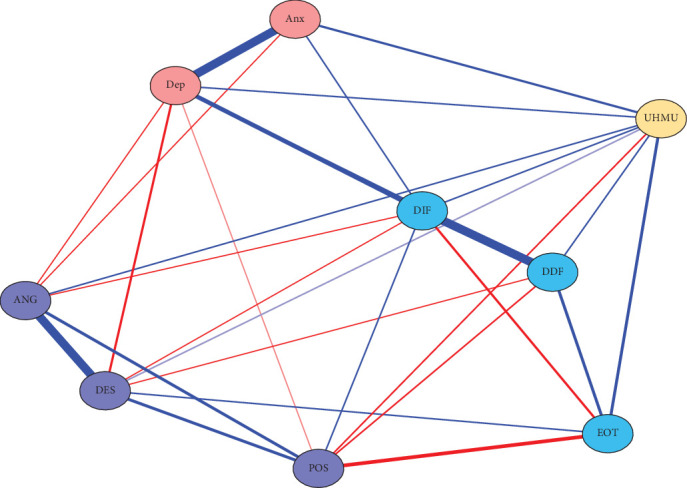
The regularized partial correlation network of unhealthy music use.

In the RPCN of unhealthy music use (Figure 6), unhealthy music use was negatively correlated with expressing positive affect in emotion regulation self-efficacy and positively correlated with anxiety, depression, difficulty identifying feelings, difficulty describing feelings, and externally oriented thinking. The three dimensions of emotion regulation self-efficacy were positively correlated. In alexithymia, externally oriented thinking was negatively correlated with difficulty identifying feelings and positively correlated with difficulty describing feelings, and difficulty describing feelings was positively correlated with difficulty describing feelings. Expressing positive affect in emotion regulation self-efficacy was positively correlated with difficulty identifying feelings and negatively correlated with externally oriented thinking and difficulty describing feelings in alexithymia. Managing anger-irritation in emotion regulation self-efficacy was negatively correlated with anxiety and depression.

**Figure 7:**
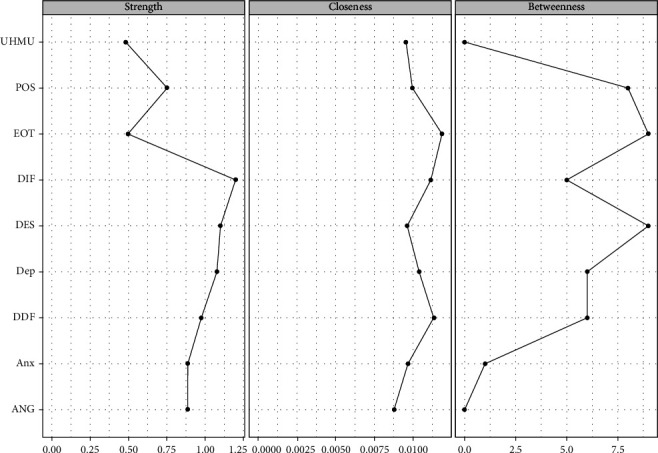
Node centrality indices of the regularized partial correlation network of unhealthy music use.

Figure 7 shows the node centrality indices of the RPCN of unhealthy music; the top three nodes of strength were difficulty identifying feelings, managing despondency-distress, and depression. The top three nodes of closeness were externally oriented thinking, difficulty identifying feelings, and difficulty describing feelings. The top three nodes of betweenness were managing despondency-distress, externally oriented thinking, and expressing positive affect.

**Figure 8:**
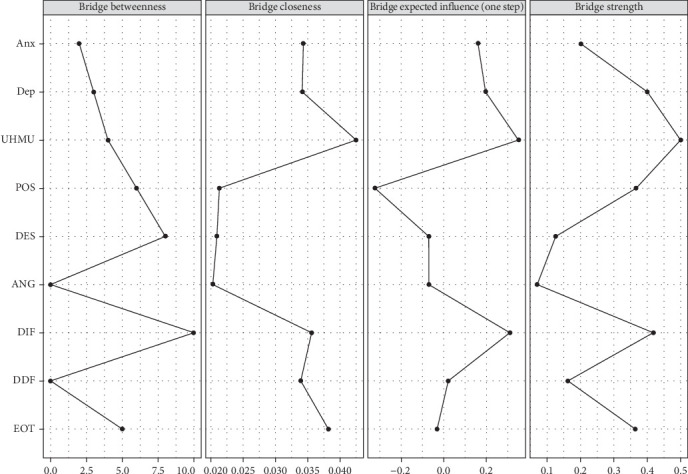
Bridge centrality indices of the regularized partial correlation model network of unhealthy music use.

Figure 8 shows the bridge centrality indices of the RPCN of unhealthy music use; the top three nodes of bridge strength were unhealthy music use, difficulty identifying feelings, and depression. The top three nodes of bridge closeness are unhealthy music use, externally oriented thinking, and difficulty identifying feelings. The top three nodes of the bridge betweenness were managing despondency-distress, difficultly identifying feelings, and externally oriented thinking. The top three nodes of the expected influence (one-step) were unhealthy music use, difficulty identifying feelings, and depression.

The CS coefficients of the bootstrapping test results for node centrality indices were strength (CS (cor = 0.7) = 0.75), closeness (CS (cor = 0.7) = 0.75), and betweenness (CS (cor = 0.7) = 0.52). The strength and closeness reached the highest CS-C level of 0.75, and the betweenness was higher than the proximity of 0.5, all indicating that the network is relatively stable [41, 42]. The figure of centrality stability and that of bootstrapped confidence intervals of estimated edge weights are attached in the supplementary materials (*Supplementary 4*: Stability of the centrality metrics of the RPCN of unhealthy music use. *Supplementary 5*: Bootstrapped confidence intervals of estimated edge-weights for the RPCN of unhealthy music).

#### 3.4.2. The Bayesian Network of Unhealthy Music Use

**Figure 9:**
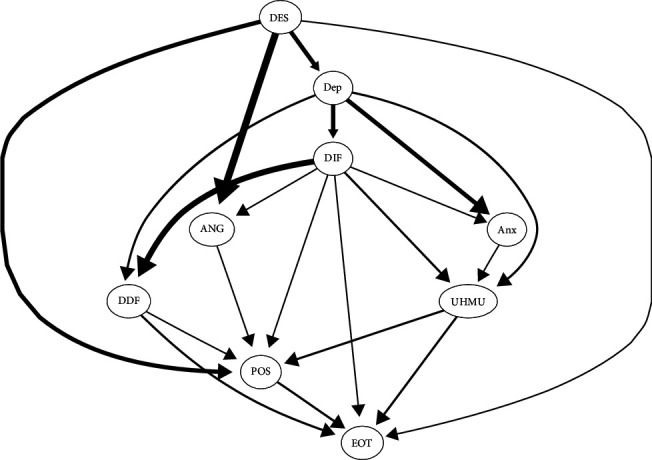
The averaged bootstrapped Bayesian network of unhealthy music use. This figure shows the averaged Bayesian network obtained by sampling the data used by the Bayesian network of unhealthy music use. In all the Bayesian networks formed by bootstrapped sampling data, the probability of edge occurrence in the figure is over than 85% and the probability of edge direction over than 50%. The thickness of the edges represents the strength of the influence between nodes; that is, the thicker the edge line, the greater the degree of influence between nodes. These edges are sorted from high to low according to their influence strength.

The averaged bootstrapped Bayesian network of unhealthy music use (Figure 9) has the following characteristics: managing despondency-distress was at the top of the network. The nodes that can be directly influenced by managing despondency-distress were expressing positive affect, managing anger-irritation, depression, and externally oriented thinking. Unhealthy music use was downstream in the network; it was influenced by depression, anxiety, and difficulty identifying feelings and can also influence externally oriented thinking. Managing despondency-distress affected difficulty identifying feelings through depression, then affected unhealthy music use, and finally influenced externally oriented thinking.

**Figure 10:**
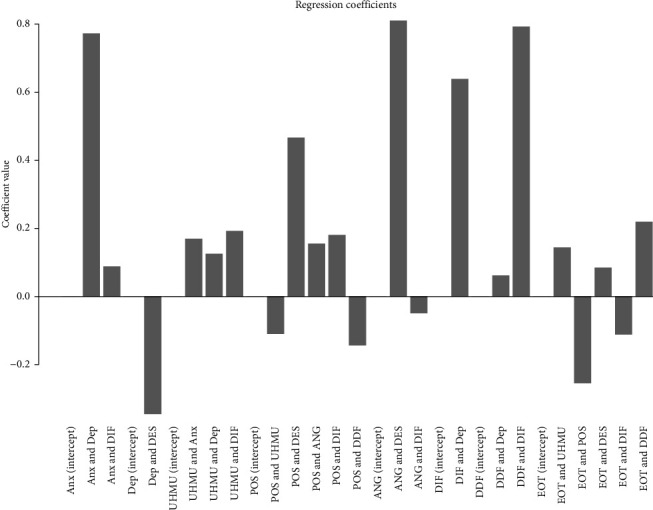
The regression coefficients of each child node in bootstrapped averaged Bayesian network of unhealthy music use.

Figure 10 shows the regression coefficients of each child node within averaged bootstrapped Bayesian network of unhealthy music use; the figure has the following characteristics: depression was negativily predicted by managing despondency-distress. Anxiety was positively predicted by depression and difficultly identifying feelings. Unhealthy music use was positively predicted by difficulty identifying feelings, anxiety, and depression. Externally oriented thinking was positively predicted by unhealthy music use, managing despondency-distress, and difficulty describing feelings and negatively predicted by expressing positive affect and difficulty identifying feelings. You can also see more details of the linear model in the supplementary materials (*Supplementary 6*: The regression coefficients of each child node in the averaged bootstrapped Bayesian network of unhealthy music use).

## 4. Discussions

In the RPCN of healthy music use (Figure 1), healthy music use was a bridge node in the network, connecting the clusters of emotion regulation self-efficacy and alexithymia. This implies that healthy music use may act as a mediator or moderator between these two constructs and that interventions that target healthy music use may have an impact on both emotion regulation and alexithymia. Moreover, healthy music use was positively correlated with expressing positive affect in emotion regulation self-efficacy and negatively correlated with externally oriented thinking in alexithymia. This suggests that healthy music use may enhance one's ability to expressing positive affect and reduce one's tendency to focus on external rather than internal cues for emotional awareness. The positive impact of healthy music use on expressing positive affect is consistent with the previous study [52].

The averaged bootstrapped Bayesian network of healthy music use (Figure 4) unraveled the intricate relationship between emotional problems, healthy music use, and emotion regulation self-efficacy. As we can see, managing despondency-distress occupied a pivotal position within the network, directly influencing depression, externally oriented thinking, managing anger-irritation, and expressing positive affect. Its cascading effect on alexithymia symptoms—difficulty identifying and describing feelings—via depression highlights its profound influence on emotional understanding and expression. In particular, healthy music use was located downstream of the network, influenced by managing anger-irritation and expressing positive affect in emotion regulation self-efficacy. Its potential to mitigate externally oriented thinking suggests a promising avenue to leverage music as a therapeutic tool to redirect cognitive patterns influenced by negative emotions [53]. The regression coefficients of the averaged bootstrapped Bayesian network of healthy music use (Figure 5) further illuminated the predictive roles within the network. Managing anger-irritation and expressing positive affect emerged as positive predictors of healthy music use, implying that these two dimensions of emotion regulation self-efficacy may serve as catalysts for engaging in adaptive music use behaviors.

In the RPCN of unhealthy music use (Figure 6), unhealthy music use demonstrated correlations with emotion regulation self-efficacy and emotion symptoms. The negative correlation between unhealthy music use and expressing positive affect in emotion regulation self-efficacy suggested a potential link between maladaptive music engagement and the ability to regulate emotion positively. This negative association highlights the possibility that unhealthy music use might inhibit one's capacity to express positive affect effectively. Furthermore, the positive correlations observed between unhealthy music use and anxiety, depression, as well as difficulties in identifying and describing feelings, point toward a potential relationship between engaging in unhealthy music use and experiencing negative emotions [54], along with challenges in emotional awareness and expression. The bridge centrality indices further emphasized the importance of unhealthy music use and difficultly identifying feelings as bridge nodes, suggesting their influential roles in connecting different aspects within this network.

The averaged bootstrapped Bayesian network (Figure 9) and the regression coefficients in the context of unhealthy music use (Figure 10) offer valuable insights into the potential causal pathways and predictive relationships between specific nodes within the network, shedding light on the dynamics underlying maladaptive music engagement and its association with emotional problems and emotion regulation self-efficacy. Managing despondency-distress emerged as a central node within the network at the top and exerted influence on several downstream nodes. In particular, it directly influenced nodes such as expressing positive affect, managing anger-irritation, depression, and externally oriented thinking. The relationships observed within the network reveal a cascade effect, where managing despondency-distress influences difficulty identifying feelings through depression, subsequently affecting unhealthy music use and, finally, influencing externally oriented thinking. This intricate path highlights the interconnectedness of emotional problems, cognitive processes, and maladaptive music behaviors. One study has unveiled that young people with depression tendency may have the intention to either cope with or to change an undesirable mood, but outcomes vary depending on the strategy used, those who chose mood matching music generally leading to either maintenance of mood or feeling worse, and those listening to music that is different to the initial undesirable mood generally resulting in mood repair or a temporary change to mood [30]. The regression coefficients further elucidate the predictive relationships among these nodes. The prediction of unhealthy music use showed a complex relationship, positively associated with difficulty identifying feelings, anxiety, and depression. This suggests that difficulties identifying feelings, along with elevated anxiety and depression symptoms, could contribute to an inclination toward unhealthy music use as a coping mechanism or manifestation of emotional problems [55, 56, 57]. Externally oriented thinking in the network exhibited a multifaceted prediction pattern, positively predicted by unhealthy music use, managing despondency-distress, and difficulty describing feelings. On the contrary, it was negatively predicted by expressing positive affect and difficulty identifying feelings. A research has found that individuals with higher levels of externally oriented thinking have difficulty perceiving emotions conveyed by music [58]. This intricate pattern highlights the role of emotion regulation and cognitive processes in shaping one's tendency toward externally oriented thinking, which could serve as a coping strategy or cognitive style associated with maladaptive music engagement.

The main findings of this study are consistent with the model of emotion dysregulation, which is that emotional competence affects the choice of emotion regulation strategies. People with high emotional competence are more likely to use adaptive emotion regulation strategies to achieve beneficial regulation goals, while people with low emotional competence are more likely to adopt maladaptive regulation strategies, leading to emotion dysregulation and preventing the development of emotional competence [4]. In this study, we took emotion regulation self-efficacy as manifestation of emotional competence, healthy music use as adaptive regulation strategy, and unhealthy music use as maladaptive regulation strategy. We have found these two emotion regulation strategies were affected by emotion regulation self-efficacy, as managing despondency-distress in regulation self-efficacy was the first node of both Bayesian networks, indicating once managing despondency-distress is changed could change the other nodes within the network, including healthy and unhealthy music use. Our findings also showcased the interplay between emotion regulation self-efficacy, emotional symptoms, and music use, which may advance our understanding of the intricated dynamics underlying music engagement and its intersection with emotion regulation self-efficacy and emotional problems. Furthermore, this study highlights the importance of considering the impact of music on emotion regulation when assessing and addressing emotional issues.

Incorporating the findings of this study into daily practice offers several actionable suggestions for enhancing emotional regulation through music. Firstly, individuals can incorporate uplifting and positive music into their daily routines to enhance mood and emotional awareness. Regularly engaging in listening to music that resonates with one's emotions can facilitate better emotional understanding and expression. Combining music with other therapeutic techniques, such as mindfulness or physical exercise, can further improve emotion regulation. Creating playlists tailored to different emotional states can help shift from negative to positive emotional patterns, while limiting exposure to sad or aggressive music can prevent exacerbating negative emotions. Keeping a journal to track emotional responses to different types of music can help identify patterns and make more informed music choices. By diversifying music choices, setting intentional listening goals, and conducting regular self-assessments, individuals can better harness the power of music to support emotional well-being.

While the study provides some insights, it has limitations, particularly the exclusion of gender-specific variations, and the influence of musical learning experiences on these relationships. Future research could explore these aspects to better understand how gender differences and musical learning might impact emotion regulation through music engagement. The results of Bayesian network which is a marriage between probability theory and graphs, can be used as one possible prediction, which should be used with caution. Moreover, although HUMS captures various aspects of healthy and unhealthy music engagement, it is important to acknowledge that certain forms of music use among students may not be fully represented. Our study may not have captured all potential healthy and unhealthy emotional regulation strategies in which music is utilized.

## 5. Conclusions

This study, utilizing the RPCN and Bayesian network approach, revealed the pivotal role of healthy music use in connecting emotion regulation and alexithymia, potentially influencing both. Conversely, unhealthy music use demonstrated associations with emotion problems and hindered expressing positive affect. Managing despondency-distress emerged as a central node, profoundly impacting emotional understanding (difficultly identifying feelings) and expression (difficultly describing feelings). Regression analysis indicated a complex between unhealthy music use and emotion problems, possibly as a coping mechanism. These findings underscore the need for interventions promoting healthy music use to enhance emotion regulation and decrease emotion problems while addressing unhealthy music use to mitigate emotion problems. Understanding this dynamic may guide targeted interventions and therapies, offering pathways to improve emotion regulation self-efficacy and mental health outcomes in individuals through music engagement.

## Figures and Tables

**Table 1:** The mean and standard deviations of each measurement for the participants.

Constant variable	Total		Male		Female	
	*N*	*M* (*SD*)	*n*	*M* (*SD*)	*n*	*M* (*SD*)
The 10-item Kessler Psychological Distress Scale						
Anxiety	16,588	7.68 (2.90)	9,128	7.45 (2.96)	7,460	7.96 (2.79)
Depression	16,588	11.83 (4.47)	9,128	11.39 (4.48)	7,460	12.38 (4.39)
The Healthy-Unhealthy Music Use Scale						
UHMU	16,588	16.54 (5.94)	9,128	16.70 (6.27)	7,460	16.34 (5.50)
HMU	16,588	16.44 (4.42)	9,128	16.34 (4.69)	7,460	16.56 (4.08)
The Toronto Alexithymia Scale						
DIF	16,588	16.11 (6.31)	9,128	15.40 (6.46)	7,460	16.98 (6.01)
DDF	16,588	12.83 (3.37)	9,128	12.57 (3.41)	7,460	13.14 (3.30)
EOT	16,588	22.01 (3.15)	9,128	22.25 (3.06)	7,460	21.70 (3.23)
The Regulatory Emotional Self-Efficacy Scale						
POS	16,588	14.73 (3.41)	9,128	14.37 (3.70)	7,460	15.18 (2.95)
DES	16,588	13.00 (3.54)	9,128	13.37 (3.72)	7,460	12.55 (3.24)
ANG	16,588	13.06 (3.63)	9,128	13.48 (3.80)	7,460	12.55 (3.34)

*Note*. UHMU, unhealthy music use; HMU, healthy music use; DIF, difficulty identifying feelings; DDF, difficulty describing feelings; EOT, externally oriented thinking; POS, expressing positive affect; DES, despondency distress; ANG, anger irritation; EOT, externally oriented thinking.

**Table 2:** The abbreviations of the variables that used in the RPCN and Bayesian network analyses.

Variables	Abbreviations
Music use	
Healthy music use	HMU
Unhealthy music use	UHMU
Emotion regulation self-efficacy	
Expressing positive affect	POS
Managing despondency-distress	DES
Managing anger-irritation	ANG
Alexithymia	
Difficulty identifying feelings	DIF
Difficulty describing feelings	DDF
Externally oriented thinking	EOT
Anxiety	Anx
Depression	Dep

## Data Availability

The data that support the findings of this study are available from the corresponding author, Chen Xijing, upon reasonable request.

## Supplementary Materials

Supplementary 1 Figure 1: stability of the centrality metrics of the RPCN of healthy music use.

Supplementary 2 Figure 2: bootstrapped confidence intervals of estimated edge-weights for the RPCN of healthy music use.

Supplementary 3 Table 1: the regression coefficients of each child node in the averaged bootstrapped Bayesian network of healthy music use.

Supplementary 4 Figure 3: stability of the centrality metrics of the RPCN of unhealthy music use.

Supplementary 5 Figure 4: bootstrapped confidence intervals of estimated edge-weights for the RPCN of unhealthy music use. The caption of each table is,

Supplementary 6 Table 2: the regression coefficients of each child node in the averaged bootstrapped Bayesian network of unhealthy music use.

Supplementary 7 R codes of network analysis.
