# Supplementary figures and images for: Music's Dual Role in Emotion Regulation: Network Analysis of Music Use, Emotion Regulation Self-Efficacy, Alexithymia, Anxiety, and Depression
Source: Depress Anxiety. 2024 Jun 28;2024:1790168. doi: 10.1155/2024/1790168 (PMC11921861; doi:10.1155/2024/1790168)

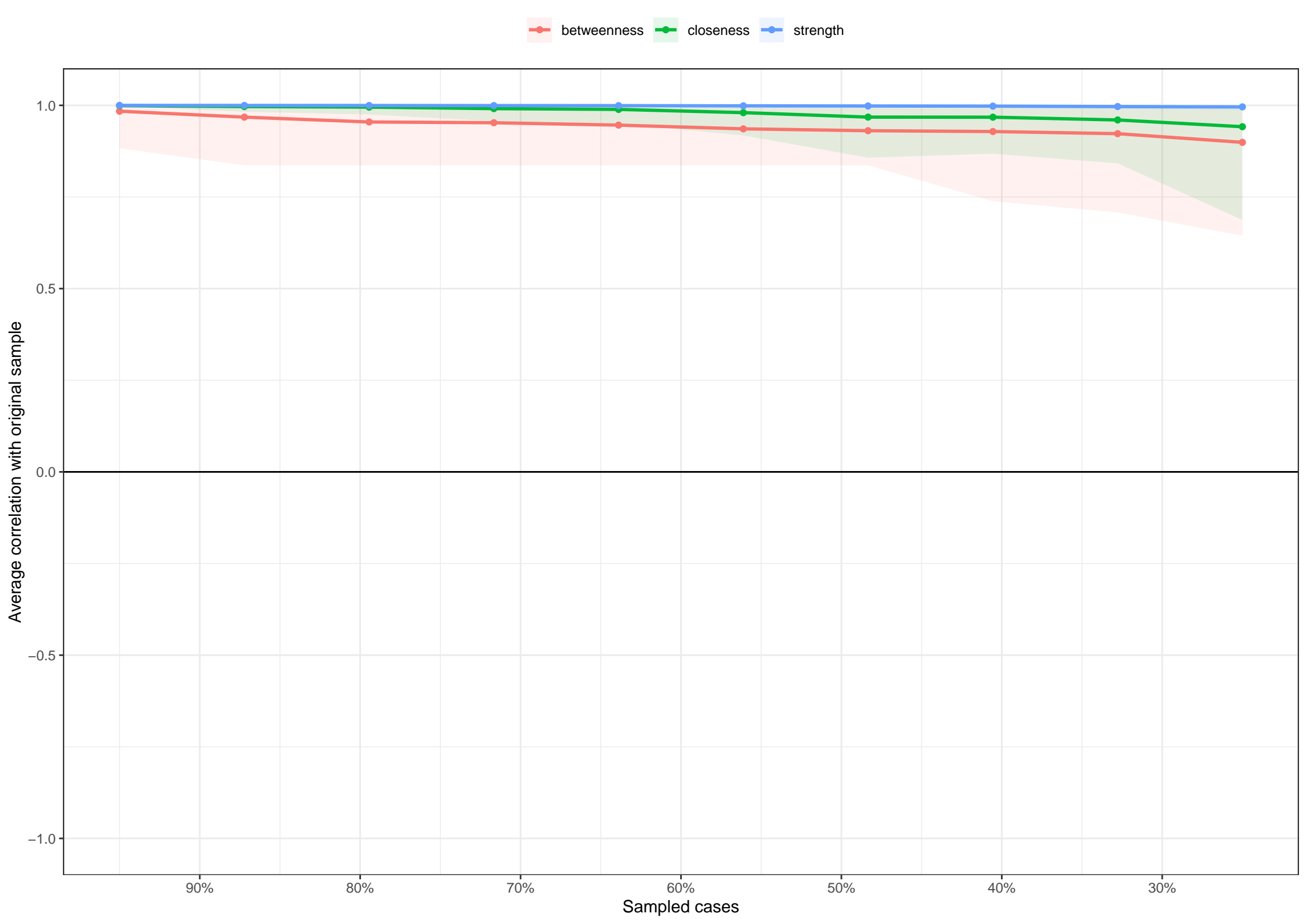

Supplement: Supplementary 1 — Figure 1: stability of the centrality metrics of the RPCN of healthy music use. [file 1790168.f1.pdf]

● Bootstrap mean    ● Sample

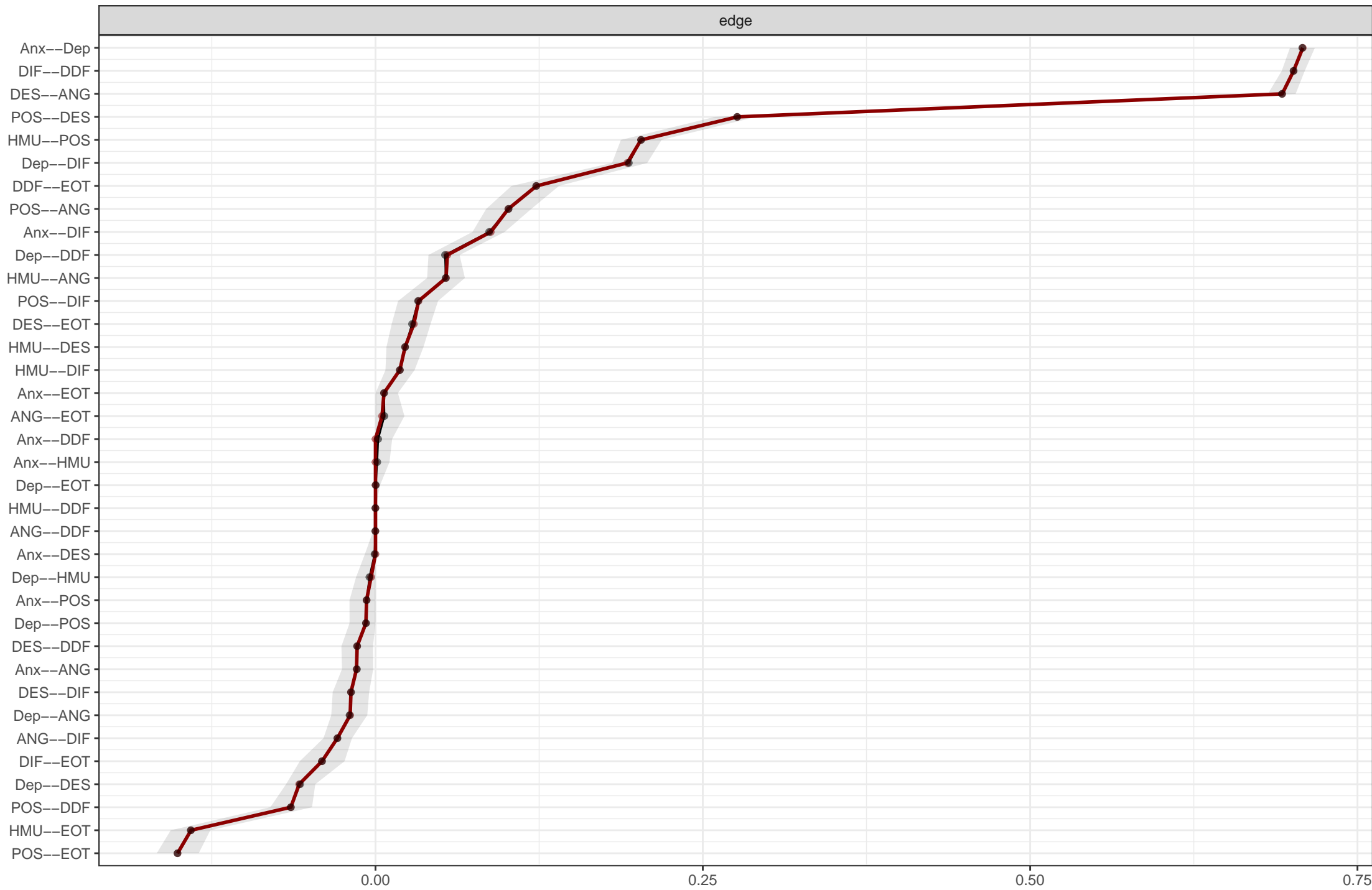

Supplement: Supplementary 2 — Figure 2: bootstrapped confidence intervals of estimated edge-weights for the RPCN of healthy music use. [file 1790168.f2.pdf]

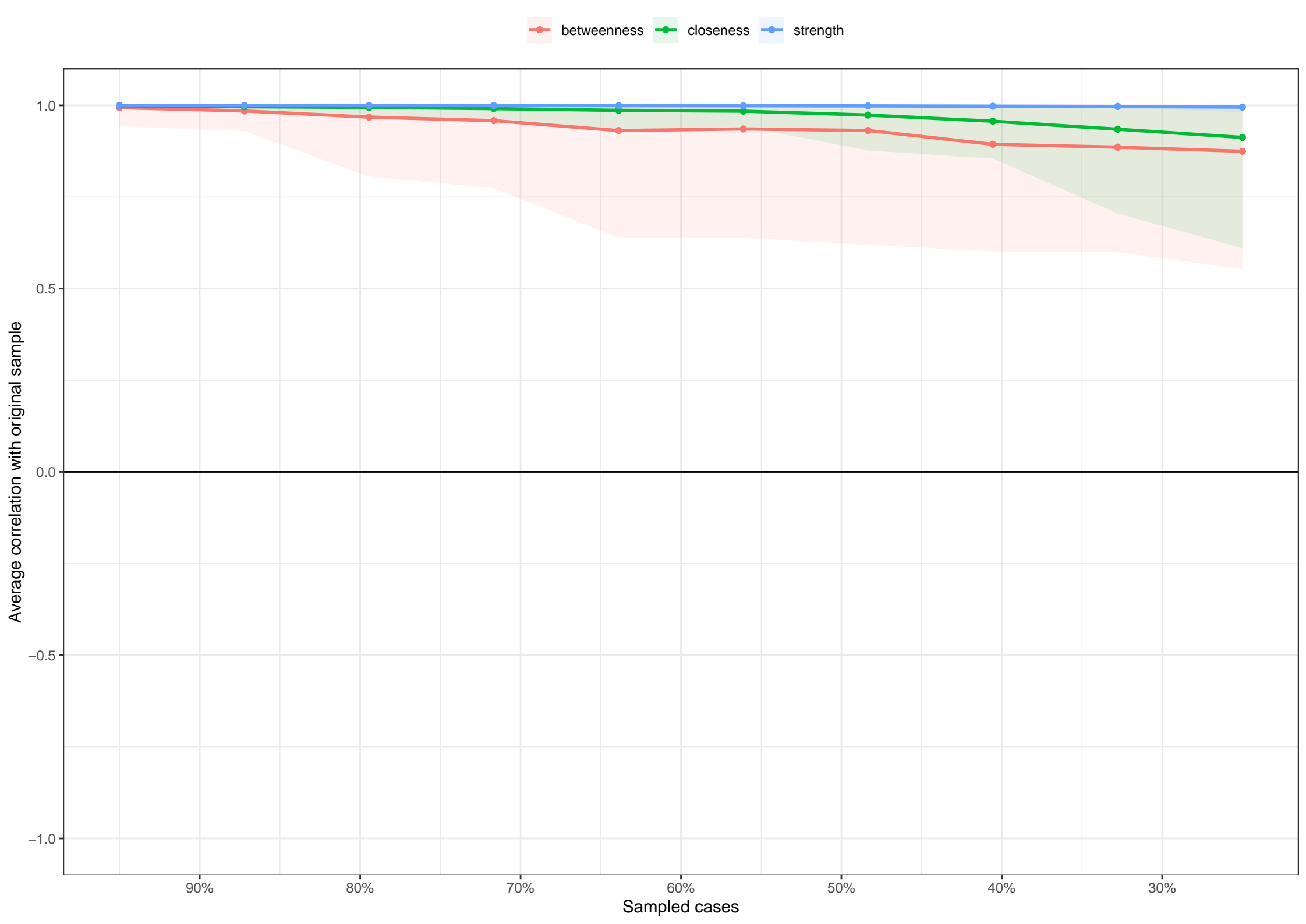

Supplement: Supplementary 4 — Figure 3: stability of the centrality metrics of the RPCN of unhealthy music use. [file 1790168.f4.pdf]

● Bootstrap mean ● Sample

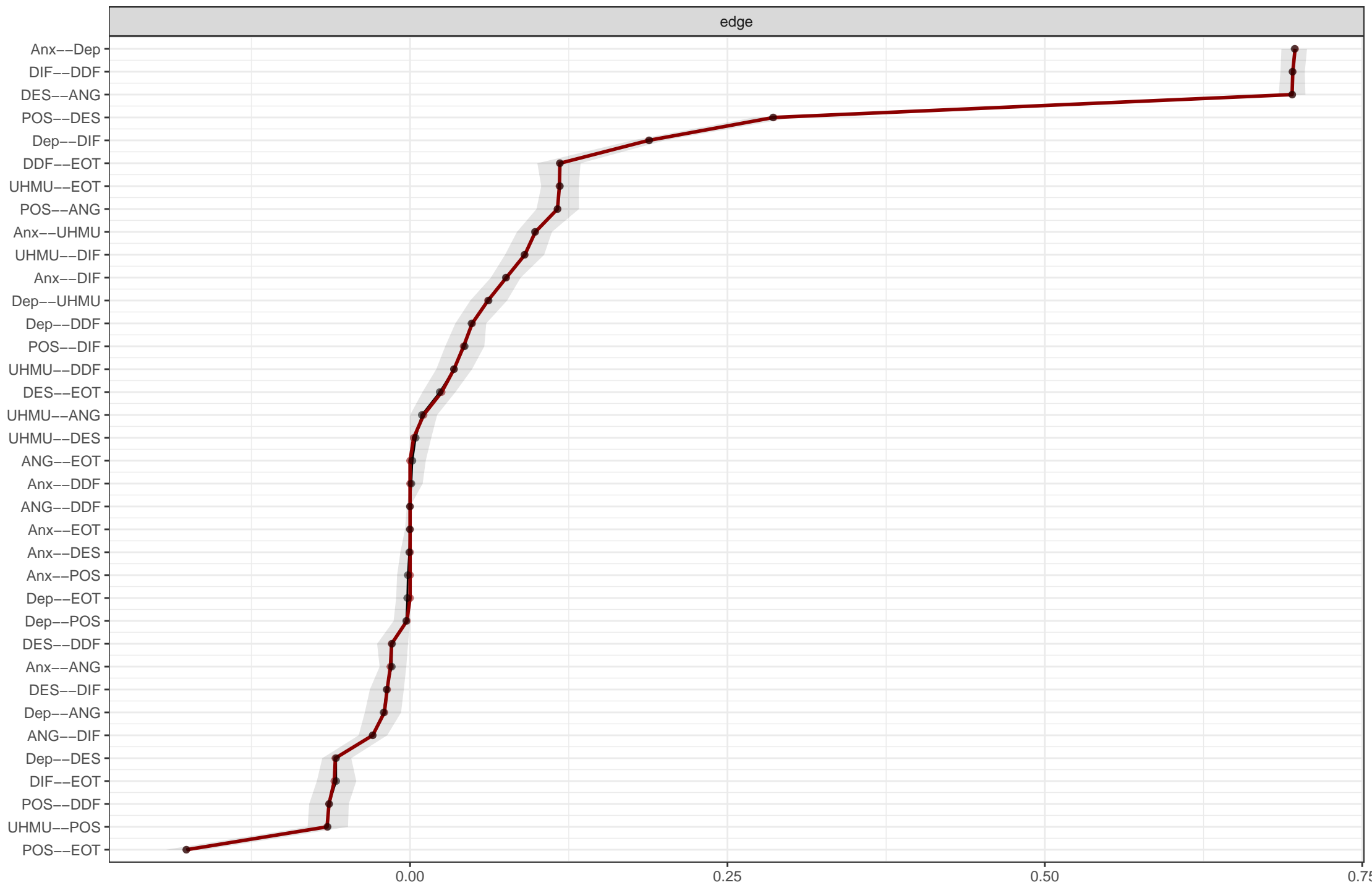

Supplement: Supplementary 5 — Figure 4: bootstrapped confidence intervals of estimated edge-weights for the RPCN of unhealthy music use. The caption of each table is, [file 1790168.f5.pdf]
