# Supplementary material for: Music's Dual Role in Emotion Regulation: Network Analysis of Music Use, Emotion Regulation Self-Efficacy, Alexithymia, Anxiety, and Depression
Source: Depress Anxiety. 2024 Jun 28;2024:1790168. doi: 10.1155/2024/1790168 (PMC11921861; doi:10.1155/2024/1790168)
Supplement: Supplementary 3 — Table 1: the regression coefficients of each child node in the averaged bootstrapped Bayesian network of healthy music use. [file 1790168.f3.pdf]

Table 1. The regression coefficients of each child node in the averaged bootstrapped Bayesian network of healthy music use.

Child nodes	Parent nodes	Total effects				Direct effects			
		R^2	RSE	F	P		β	SE	P
Anx	Dep+ DIF	0.69	0.56	18620	< 0.001	Dep	0.77	0.01	< 0.001
						DES	0.09	0.01	< 0.001
Dep	DES	0.12	0.94	2241	< 0.001	intercept	0.00	0.01	1
						DES	-0.35	0.01	< 0.001
HMU	POS + ANG	0.12	0.94	1266	< 0.001	intercept	-0.00	0.01	1
						POS	0.29	0.01	< 0.001
						ANG	0.10	0.01	< 0.001
POS	DES+ANG+DIF	0.37	0.80	2077	< 0.001	intercept	0.00	0.01	1
						DES	0.48	0.01	< 0.001
						ANG	0.16	0.01	< 0.001
						DIF	0.02	0.01	0.000
ANG	DES+DIF	0.66	0.56	1.8	< 0.001	intercept	0.00	0.00	1
						DES	0.81	0.01	< 0.001
						DIF	-0.05	0.01	< 0.001
DIF	DEP	0.41	0.77	11450	< 0.001	intercept	0.00	0.01	1
						Dep	0.64	0.01	< 0.001
DDF	POS+DIF	0.70	0.55	18890	< 0.001	intercept	0.00	0.00	1
						Dep	0.05	0.01	< 0.001
						POS	-0.07	0.00	< 0.001
						DIF	0.79	0.01	< 0.001
EOT	HMU+POS+DES+DIF+DDF	0.12	0.94	435	< 0.001	intercept	-0.00	0.01	1
						HMU	-0.17	0.01	< 0.001
						POS	-0.23	0.01	< 0.001
						DES	0.11	0.01	< 0.001
						DIF	-0.06	0.01	0.00
						DDF	0.23	0.01	< 0.001
