# Supplementary material for: Music's Dual Role in Emotion Regulation: Network Analysis of Music Use, Emotion Regulation Self-Efficacy, Alexithymia, Anxiety, and Depression
Source: Depress Anxiety. 2024 Jun 28;2024:1790168. doi: 10.1155/2024/1790168 (PMC11921861; doi:10.1155/2024/1790168)
Supplement: Supplementary 6 — Table 2: the regression coefficients of each child node in the averaged bootstrapped Bayesian network of unhealthy music use. [file 1790168.f6.pdf]

Table 2. The regression coefficients of each child node in the averaged bootstrapped Bayesian network of unhealthy music use.

Child nodes	Parent nodes	Total effects				Direct effects			
		R^2	RSE	F	P		ß	SE	P
Anx	Dep+ DIF	0.70	0.56	18620	< 0.001	Intercept	0.00	0.00	1.00
						Dep	0.77	0.01	< 0.001
						DIF	0.09	0.01	< 0.001
Dep	DES	0.12	0.94	2241	< 0.001	Intercept	0.00	0.01	1.00
						DES	-0.35	0.01	< 0.001
UHMU	Anx+ Dep+ DIF	0.19	0.90	1178	< 0.001	Intercept	0.00	0.01	1.00
						Anx	0.17	0.01	< 0.001
						Dep	0.13	0.01	< 0.001
						DIF	0.19	0.01	< 0.001
POS	UHMU+ DES+ ANG+ DIF+ DDF	0.39	0.78	3209	< 0.001	Intercept	0.00	0.01	1.00
						UHMU	-0.11	0.01	< 0.001
						DES	0.47	0.01	< 0.001
						ANG	0.16	0.01	< 0.001
						DIF	0.18	0.01	< 0.001
						DDF	-0.14	0.01	< 0.001
ANG	DES+DIF	0.69	0.56	18000	< 0.001	Intercept	0.00	0.00	1.00
						DES	0.81	0.01	< 0.001
						DIF	-0.05	0.01	< 0.001
DIF	Dep	0.41	0.77	11450	< 0.001	Intercept	0.00	0.01	1.00
						Dep	0.64	0.01	< 0.001
DDF	Dep+DIF	0.70	0.55	12880	< 0.001	Intercept	0.00	0.00	1.00
						Dep	0.06	0.01	< 0.001
						DIF	0.79	0.01	< 0.001
EOT	UHMU+POS+DES+DIF+DDF	0.12	0.94	464	< 0.001	Intercept	0.00	0.01	1.00
						UHMU	0.14	0.01	< 0.001
						POS	-0.25	0.01	< 0.001
						DES	0.09	0.01	< 0.001
						DIF	-0.11	0.01	< 0.001
						DDF	0.22	0.01	< 0.001
