# Supplementary material for: Music's Dual Role in Emotion Regulation: Network Analysis of Music Use, Emotion Regulation Self-Efficacy, Alexithymia, Anxiety, and Depression
Source: Depress Anxiety. 2024 Jun 28;2024:1790168. doi: 10.1155/2024/1790168 (PMC11921861; doi:10.1155/2024/1790168)
Supplement: Supplementary 7 — R codes of network analysis. [file 1790168.f7.docx]

########### R codes of RPCN and Bayesian network analysis ###########

## required packages

## Rgraphviz is on BioConductor, the remaining packages on CRAN

#installing BiocManager first then Rgraphviz

#if (!require("BiocManager", quietly = TRUE)) install.packages("BiocManager") BiocManager::install(version = "3.17")

#BiocManager::install(c("Rgraphviz"))

library(dplyr)

library(readr)

library("huge") ## used for normalization transformation for GGM

library("bootnet") ## estimate network

library("qgraph") ## network plots and graphical LASSO

library("bnlearn") ## Bayesian network package

library("Rgraphviz") ## used for visualizing Bayesian networks

library("networktools") ## used for computing bridge strength

library("xtable")

## ------------------------ data import and data manipulation -------------------------------

surveydata<-read.table("C:/Users/Mailm/Downloads/Rscripts/网络分析/survey.csv",

header = TRUE, sep=",",na="NA")

#select unhealthy data

netdata<- surveydata %>% select("Anx","Dep","UHMU","POS","DES","ANG","DIF","DDF","EOT")

gnames<-c("The 10-item Kessler Psychological Stress Scale","The 10-item Kessler Psychological Stress Scale","The Healthy-Unhealth Music Use Scale","The Regulatory Emotional Self-Efficacy Scale","The Regulatory Emotional Self-Efficacy Scale","The Regulatory Emotional Self-Efficacy Scale","The Toronto Alexithymia Scale","The Toronto Alexithymia Scale","The Toronto Alexithymia Scale")

longnames<-c("Anxiety","Depression","Unhealthy Music Use", "Expressing Positive Affect","Despondency-distress","Anger-irritation","Difficulty Identifying Feelings","Difficulty Describing Feelings","Externally-Oriented Thinking")

#select healthy data

netdata<- surveydata %>% select("Anx","Dep","HMU","POS","DES","ANG","DIF","DDF","EOT")

gnames<-c("The 10-item Kessler Psychological Stress Scale","The 10-item Kessler Psychological Stress Scale","The Healthy-Unhealth Music Use Scale","The Regulatory Emotional Self-Efficacy Scale","The Regulatory Emotional Self-Efficacy Scale","The Regulatory Emotional Self-Efficacy Scale","The Toronto Alexithymia Scale","The Toronto Alexithymia Scale","The Toronto Alexithymia Scale")

longnames<-c("Anxiety","Depression","Healthy Music Use", "Expressing Positive Affect","Despondency-distress","Anger-irritation","Difficulty Identifying Feelings","Difficulty Describing Feelings","Externally-Oriented Thinking")

##1. the regularized partial correlation network

netdata<-huge.npn(netdata) #data normalization for GGM

cormat <- cor_auto(netdata)

glassoFit <- EBICglasso(cormat, n = nrow(netdata))

node_colors <- c("#FC9998","#FC9998","#FFE779","#647FD5","#647FD5","#647FD5","#6FCBE3","#6FCBE3","#6FCBE3")

glassoNet <- qgraph(glassoFit, layout = "spring",color=node_colors,

groups = gnames,nodeNames=longnames,

cut=0.01,vsize2 = 0.03,vsize = 6,legend.cex=.3,

labels = colnames(netdata),posCol="#1772FF",negCol="red",

legend=FALSE)

## 1.1. plot the node centrality indices( z-standardization)

centralityPlot(glassoNet,include =

c("Strength","Closeness","Betweenness"))

#1.2. estimate bridge centrality values for each node

mynetwork <- estimateNetwork(netdata, default = "EBICglasso",

corMethod="cor",

corArgs=list(method="spearman",

use="pairwise.complete.obs"))

myplot<-plot(mynetwork,layout = "spring",groups=gnames)

mybridge<-bridge(myplot,communities = gnames,

useCommunities="all",

directed=NULL,nodes=NULL) # For all communities, or specify the communities

plot(mybridge,include=c("Bridge Strength","Bridge Closeness","Bridge Betweenness", "Bridge Expected Influence (1-step)"))

##1.3. boostrap the regularized partial correlation network

glassoFit2 <- estimateNetwork(netdata, default = "EBICglasso") ## re-estimate network

##1.4. edge weight accuracy

set.seed(123)

glassoBoot1 <- bootnet(glassoFit2, statistics = c("edge","Strength","Closeness","Betweenness"),nBoots = 1000, nCores = 4)

summary(glassoBoot1)

plot(glassoBoot1,statistics = c("edge"),labels=TRUE,order="sample") ## plot edge differences (y-axis)

## The red line indicates the sample values and the gray area the bootstrapped CIs.

## Each horizontal line represents one edge of the network, ordered from

## the edge with the highest edge-weight to the edge with the lowest edge-weight.

## We see which edge weights differ significantly from each other.

## 1.5. Bootstrapped difference tests between edge-weights that were non-zero

## In the estimated network (first plot) and node centrality of the symptoms (second and after plots):

plot(glassoBoot1, "edge", plot = "difference", onlyNonZero = TRUE, order = "sample")

plot(glassoBoot1, "strength", plot = "difference", order = "sample")

plot(glassoBoot1, "Closeness", plot = "difference", order = "sample")

plot(glassoBoot1, "Betweenness", plot = "difference", order = "sample")

## Gray boxes indicate nodes or edges that do not differ significantly from one another and

## black boxes represent nodes or edges that do differ significantly from one another.

## Note: white boxes in the second and after plots show the value of node strength.

## 1.6. centrality stability

set.seed(123)

glassoBoot2 <- bootnet(glassoFit2, statistics = c("Strength","Closeness","Betweenness"),nBoots = 1000, type = "person", nCores = 4)

plot(glassoBoot2,statistics = c("Strength","Closeness","Betweenness"))

corStability(glassoBoot2)

## Average correlations between centrality indices of networks sampled with persons

## dropped and the original sample. Lines indicate the means and areas indicate the range

## from the 2.5th quantile to the 97.5th quantile

## 2.Constuct Bayesian network

netdata <- as.data.frame(apply(netdata, 2, as.numeric))

set.seed(123)

## 2.1. Fit a first Bayesian network, based on 50 random re-starts and 100 perturbations for each re-start.

# constuct the Bayesian network with hc algorithm

#fitBN1 <- hc(netdata, restart = 50, perturb = 100) ## hc gives directed graph

#bnlearn::score(fitBN1, data = netdata) ## global network score

#astr <- arc.strength(fitBN1, netdata,"bic-g") ## connection strength

#astr[order(astr[,3]), ] ## sorted edge strength from strongest to weakest

#strength.plot(fitBN1, astr, fontsize=15,shape = "ellipse") ## plot the net

## compute the conditional desyties of each node

#fit<-bn.fit(fitBN1,netdata)

#fit

#coefficients(fit) ## print the conditional desyties of each node

## 2.2.stabilize the network across multiple samples through bootstrapping:

BST <- boot.strength(netdata, R = 1000, algorithm = "hc",

algorithm.args = list(restart = 5, perturb = 10), debug = TRUE)

## filter the ones with a strength larger than 0.85 and a direction probability > 0.5

BST[BST$strength > 0.85 & BST$direction > 0.5, ]

## strength: connection strength, e.g. 0.85 means that this connection appears in 85% of the fitted networks.

## direction: probability of the direction, e.g. 0.5 means that in 50% of the fitted networks the connection goes in the direction depicted in the graph.

avgnet1 <- averaged.network(BST, threshold = 0.85)

bnlearn::score(avgnet1, data = netdata)

astr1 <- arc.strength(avgnet1,netdata, "bic-g") # compute edge strengths

strength.plot(avgnet1, astr1, fontsize=12,shape = "ellipse") #plot net

###### 3.Visualize Regression Coefficients ######

# Extract the structure of the network

network_structure <- amat(avgnet1)

# Get the names of nodes in the network

node_names <- nodes(avgnet1)

# Perform linear regressions for each node based on its parents

regression_coefficients <- list()

regression_results <- list()

for (node in node_names) {

# Find parents of the current node

parents <- parents(avgnet1, node)

# Check if the node has parents

if (length(parents) > 0) {

# Create a formula for regression

formula <- as.formula(paste(node, "~", paste(parents, collapse = " + ")))

# Fit a linear regression using lm() function

regression_model <- lm(formula, data = netdata)

# Store regression coefficients

regression_coefficients[[node]] <- coef(regression_model)

regression_summary<- summary(regression_model)

regression_results[[node]] <- regression_summary

} else {

# If the node has no parents, store a message indicating no regression is performed

regression_coefficients[[node]] <- "No parents - no regression performed"

}

}

# Print or access regression coefficients for each node

print(regression_coefficients)

print(regression_results)

# Extract coefficients and p-values for the selected nodes

coefficients <- lapply(regression_results, function(result) {

if (!inherits(result, "character")) {

return(data.frame(result$coefficients[, c("Estimate", "Pr(>|t|)")]))

} else {

return(NULL)

}

})

coefficients <- coefficients[sapply(coefficients, function(x) !is.null(x))]

# Combine coefficients into one data frame

combined_table <- do.call(rbind, coefficients)

# Print the combined table using xtable (LaTeX format)

# Assuming 'combined_table' contains your coefficients and p-values

# Extract coefficients and p-values

coefficients <- combined_table[, "Estimate"]

p_values <- combined_table[, "Pr...t.."]

#Create a wider plot area to accommodate longer node names

par(mar = c(7, 5, 2, 1)) # Adjust the margins as needed

# Create a bar plot for coefficients with wider space for node names

barplot(coefficients, main = "Regression Coefficients", ylab = "Coefficient Value",

names.arg = rownames(combined_table), las = 2, cex.names = 0.8)
